# Deafening silence on a vital issue: The World Health Organization has ignored the sexuality of persons with disabilities

**DOI:** 10.4102/ajod.v7i0.474

**Published:** 2018-07-16

**Authors:** Christine Peta

**Affiliations:** 1 Centre for Rehabilitation Studies, Department of Global Health, Faculty of Medicine and Health Sciences, Stellenbosch University, South Africa

## Abstract

In 2016, the World Health Organization, through the Global Cooperation on Assistive Technology Initiative, issued the Priority Assistive Products List which is meant to be a guide to member states of the 50 assistive products needed for a basic health care and/or social welfare system; it is also a model from which nations can develop their national priority assistive products lists. The aim of this opinion paper is to share my views about the Priority Assistive Products List on the grounds that it makes no distinct mention of sexual assistive devices, yet research has indicated that sexuality is an area of great concern for persons with disabilities. In any case, sexuality forms a core part of being human, and it impacts on both the physical and mental well-being of all human beings. I conclude in part that, in its present format, the list perpetuates the myth that persons with disabilities are asexual beings who are innocent of sexual thoughts, feelings and experiences. The list also propagates the stereotype that sexuality is a sacred, private, bedroom matter that should be kept out of the public domain, to the detriment of the health and well-being of persons with disabilities.

## Sexuality, assistive products list and assistive devices

Sexuality encompasses the total behaviour of who we are as human beings, from birth to death, including the growth of our bodies in relation to, among other things, puberty, menstruation and reproduction; it involves how a person feels about himself or herself, how he or she feels about others, as well as his or her interaction with society (Makinwa-Adebusoye & Tiemoko 2007). People may explore their bodies through masturbation or personal fantasies at the individual level, but they may still want to choose persons with whom they address their sexuality with. However, the World Health Organization (WHO) Assistive Products List (APL) is devoid of sexual assistive devices, thereby ignoring the sexuality of persons with disabilities and reducing complex beings to a single social life attribute of disability. Yet, the American Occupational Therapy Association asserts that sexuality is part of every person’s life and therefore an activity of daily living along with eating, toileting and dressing (Naphtali, MacHattie & Elliott 2009); Gomez (2012) and McKenzie (2012) also assert that no human being is asexual.

Nevertheless, the ability of persons with disabilities to engage in sexual activity can to a great extent be altered by motor, sensory and autonomic dysfunction (Naphtali et al. 2009). Motor dysfunction may relate to the movement of arms and legs, sensory dysfunction to temperature and touch sensations, and autonomic dysfunction to the regulation of blood pressure. However, persons with disabilities have commonly reported that gaining or regaining sexual functioning is one of their priorities (Anderson 2004; Peta 2017a). A study carried out by Anderson (2004) in the United States revealed that recovering sexual function tops the list of priorities of persons with paraplegia. Whilst the disability movement prioritises issues such as housing and transportation, Waxman (1989:2) states that ‘many people with disabilities consider sexuality to be an area of their greatest oppression: We are more concerned with being loved and finding sexual fulfilment than getting on a bus’. The irony of the matter is that compared to other rehabilitative spheres, such as occupational therapy and physiotherapy, sexual rehabilitation receives the least attention, yet a part of the United Nations Convention on the Rights of Persons with Disabilities in Article 26 (United Nations 2006), directs state parties to design and implement comprehensive rehabilitation services.

Some scholars have noted that the subject of sexuality is regarded as a very sensitive topic which is difficult to discuss even among clinicians (Mall & Swartz 2012); it is therefore not surprising that the stakeholders who participated in the formulation of the APL may have found it difficult to advocate for the inclusion of sexual assistive devices. However, in this opinion paper, the subject of sexuality is closely linked to assistive devices; hence, the meaning of assistive devices is explained below.

Under the WHO Global Cooperation on Assistive Technology (GATE) initiative, a list of 50 assistive products, namely, the APL was published. The main goal of the GATE project is to improve on the status quo, where currently only 1 person among 10 people of those in need is able to access assistive products. Assistive devices or assistive products are defined as:

Any external product (including devices, equipment, instruments or software), especially produced or generally available, the primary purpose of which is to maintain or improve an individual’s functioning and independence, and thereby promote their well-being. Assistive products are also used to prevent impairments and secondary health conditions. (WHO 2016:1)

In line with the above definition, the significance of sexual assistive devices in preventing impairments and secondary health conditions as well as promoting the well-being of persons with disabilities is further discussed below.

## Ethical consideration

The confidentiality and anonymity of persons who took part in other studies that were undertaken by the author of this opinion paper and that are referenced in this publication are upheld.

## The assistive products list is silent on sexual assistive devices

The most common examples of assistive devices that have been identified by WHO include hearing aids, communication boards, wheelchairs, canes, prosthetic and orthotic devices, spectacles, low vision aids, portable ramps and incontinence products (absorbent) (AFRINEAD n.d.; WHO 2016). In Canada, Naphtali et al. (2009) illuminated examples of sexual assistive devices that may enhance the sexual expression of persons with disabilities; intimate riders, harnesses, leather cuffs, hands free kits, massagers and body bouncers. However, articulating a comprehensive list of such devices and the distinct ways in which they enhance the sexuality of persons with disabilities or promote their well-being would take this opinion paper beyond its requirements in terms of both scope and length. Nevertheless, the intimate rider, which was designed by an individual with C6-7 quadriplegia to facilitate varied positions of sexual activities (Naphtali et al. 2009), is an example of one of the sexual assistive devices that could have been included in the APL. The rider comprises accessible supports such as grab bars or counter tops; its use along with additional sexual assistive devices could promote the health and well-being of persons with disabilities as illustrated in the example below.

A study carried out in Zimbabwe by Peta (2017a; 2017b) revealed that women who acquire physical impairment in the course of their marriages, particularly spinal cord–induced impairment, are often deserted by their husbands who castigate them for the ways that disability alters their sexual expression, thereby withdrawing all forms of support that would have been extended to the wife before the ‘arrival’ of the disability. The result is untold anguish, which among other impairments results in the occurrence of psychosocial impairments that include bipolar affective disorder or the occurrence of disease and impairments among children. The women struggle to economically fend for themselves and their minor children, whilst at the same time making efforts to adjust to the social and sexual challenges that are brought about by disability (Peta 2017a; 2017b). The use of the intimate rider and additional assistive sexual devices could reduce sexual frustration, save marriages and keep nuclear families together within contexts where healthy children are likely to be raised. Nevertheless, the omission of sexual assistive devices in the APL can be attributed to the five-stage strategy which guided the formulation of the APL (AFRINEAD n.d.), as further discussed below.

The APL emerged from a scoping review, pilot survey, Delphi exercise, global survey and global consultation (AFRINEAD n.d.). The process was also guided by an initial proposed breakdown of 155 ‘important’ assistive products that were from the onset solicited to fit in six pre-determined areas: (1) mobility, (2) vision, (3) hearing, (4) communication, (5) cognition and (6) environment. Sexuality could have been included as a seventh area, considering that all human beings including persons with disabilities are sexual beings; disability intersects with sexuality which needs to be supported by appropriate assistive sexual devices. That is not to dispute the fact that the APL is a guide, but it is to say that as evidenced by the earlier example of women with disabilities in Zimbabwe, a deafening silence about sexuality in such an original international list makes it easier to keep the subject of sexuality ‘hidden’ in subsequent national lists, thereby opening doors for sexuality to be used negatively in abuse, control, oppression, to cover up sexual scandals and to misinform one another (Interesting Interests 2009; Peta 2017a); disability adds a rung to the ladder of such vulnerabilities.

In any case, some studies undertaken in both the Global North and the Global South have confirmed the need for people with disabilities to have appropriate sexual assistive devices that enable them to have a positive sexual life, which ultimately enhances their health and well-being (Mackelprang 2009; Naphtali et al. 2009; Peta 2017a; Taylor 2011). In the United States, Mackelprang (2009) points at vibrators and states that such sexual assistive devices are valuable because they enhance stimulation when there is reduced sensation and they can also be useful in instances where mobility is restricted. In the United Kingdom, Taylor (2011) calls upon occupational therapists to pay attention to the impact of assistive devices on sexual expression. A study carried out in Zimbabwe by Peta (2017a, 2017b) revealed emotional distress among women with disabilities if something goes wrong during intimate moments with their male partners, whilst the women are using inappropriate assistive devices or none at all owing to unavailability. The use of appropriate sexual assistive devices contributes to the promotion of safe sexual environments, safe sexual practices, keeping families together and the raising of healthy children whose survival, protection and development are prerequisites for future development and humanity.

## Conclusion

There is need to bring the subject of sexuality into the discourse of assistive devices; persons with disabilities are not asexual beings. I therefore call upon the architects of the APL to come to terms with the fact that people with disabilities have a right to sexual expression, and as noted by Morris (2001) they should be allowed access to relevant entitlements (including sexual assistive devices), not only because they are human beings but also because they are equal citizens. The pledge that ‘no one will be left behind’ and that governments will endeavour to reach ‘the furthest behind first’ lies at the heart of the Sustainable Development Goals (SDGs) (United Nations Development Program 2018). To ensure that no one is left behind, architects of the APL should include sexual assistive devices that are not exclusionary but that embrace persons with disabilities of all sexual orientations, including lesbian, gay, bisexual, transgender and intersex (LGBT1).

Perpetual failure to openly name assistive devices that enhance the sexual expression of persons with disabilities may result in the APL sending a potent message which may be misconstrued to mean that the subject is an unimportant side issue. Such distinct naming promises to go a long way in raising awareness of the sexual rights of persons with disabilities and to nurture receptiveness to their rights. Whether we like it or not, sexuality is a major rehabilitation priority for persons with disabilities; hence, it should be respected, celebrated and be openly talked about (Naphtali et al. 2009). Sustainable Development Goal Number 3 calls upon state parties to ‘ensure healthy lives and promote well-being for all at all ages’, but how can SDG Number 3 be attained if the APL maintains a deafening silence on assistive devices that are directly related to sexual rehabilitation? The open inclusion of sexual language in international policy documents, legal instruments and guidelines can go a long way in dismantling myths that surround the sexuality of persons with disabilities, thereby enhancing their quality of life, health and well-being.
